# Books and Pamphlets Received

**Published:** 1888-01

**Authors:** 


### BIBLIOGRAPHY.

A PRACTICAL TREATISE on the Diseases of the Hair and Scalp. By Geo. Thomas Jackson, M. D., Instructor in Dermatology in the New York Polyclinic; Assistant Visiting Physician to the New York Skin and Cancer Hospital; Member of the New York Dermatological Society, etc., New York. E. B. Treat & Co., 771 Broadway; 1887 Cloth; 326 pages, price, $2.75. Illustrated.

Of late years diseases of the hair and scalp have, for some reason, become more numerous, or have seemingly become more numerous, from the fact that they are receiving more attention than formerly; they are being studied and treated scientifically, as they should be, instead of overlooked or slighted, or, treated empirically, as formerly, and Dr. Jackson has earned the thanks of the medical profession by this clear and concise treatise on a subject as little understood by the profession as perhaps any branch of medical study.

The work before us, which, by the bye, does credit to the publishers, especially as it is somewhat out of their line, we believe, to issue medical books, treats of all the common forms of the diseases to which the hair and scalp are subject, and gives, besides, accounts of anomalies, and of remarkable cases.

The first and second chapters are devoted to the anatomy and physiology of the hair, and the third to its hygiene, a chapter which could be read with profit by every person in the land.

Dr. Jackson's access to the great skin hospitals of New York, and his connection with the Polyclinic, give him unusual opportunities for seeing and studying these diseases in all their phases. The work is an acceptable addition to our library.

---

WHAT TO DO IN CASES OF POISONING, by Wm. Merrell, M. D., F. R. C. P., Lecturer on Pharmacology and Therapeutics in the Westminster Hospital, Examiner in Materia Medica in the University of Edinburgh and in the Royal College of Physicians of London, Eng. First American from the fifth English edition. Edited by Frand Woodbury, M. D., Fellow of the College of Physicians of

Philadelphia, etc., etc. Published by the Medical Register Co., Philadelphia.

This little work has been a success; it has met a large sale, and in England has reached its fifth edition. It is beyond doubt the best book of its kind that has appeared. Our colleague, Dr. Woodbury, has rendered a service to the profession of America in bringing it out; he struck a popular chord, and, judging by what the author says of the "fee" for attending cases of poisoning, it would be worth one's while to become an expert. He says, "according to the circumstances and the social position of the patient the 'Specialist' (in poisoning cases!) is entitled to charge from $125 to $250." It is a wonder that we do not hear of "specialists for poisoning cases,' and presume that it is amongst the probabilities of the near future.

---

ATHOSIS; a Satire on Modern Medicine, by Thomas C. Minor; 12 mo., p. 194; Cincinnati, R. Clark & Co., 1887.

We have been in possession of this little work some time, and have seriously tried to read it. It is a novel, written in an airy, off-hand style, and is we suppose, what it pretends to be, a clever satire. It certainly makes some hard hits at a certain class of practitioners of modern times. There is a deal of humor in it, and it will amuse one if he has no more serious business on hand.

---

THE MEDICAL WORLD VISITING LIST. Being a Daily Record of Practice and Accounts, without the use of Signs, and hence, need no Transferring. Arranged in removable Tablets. The Medical World, 1520 Chestnut St., Philadelphia.

We have received one of the first of this series, and are pleased with it in some respects; the arrangement *would be* a great convenience to physicians if the book were larger; the objection is that the space is so small and the lines so close together that one can with difficulty make any intelligible entry of any kind. It is a good idea to have removable tablets, but it seems to us that if one has much practice he would have to keep a stock of tablets, one for each patients account. Price $1.50.

---

DISEASES OF THE FEMALE MAMMARY GLANDS, by Th. Billroth, M. D., of Vienna, and NEW GROWTHS OF THE UTERUS, by A. Gusserow, M. D., of Berlin. These two works constitute Vol. IX. of the *Cyclopœdia of Obstetrics and Gynecology*, (12 vols., price $16.50), issued monthly during 1887. New York: William Wood & Company.

DISEASES OF THE FEMALE URETHRA AND BLADDER. By F. Winckel, M. D., of the Royal University, Munich; and DISEASES OF THE VAGINA, by A. Breisky, M. D., of the Royal University, Vienna. Edited by Egbert H. Grandin, M. D., of New York. These two treatises constitute Vol. X. of a *Cyclopœdia of Obstetrics and Gynecology* (12 vols., price $16.50), issued monthly during 1887. New York: William Wood & Company.

The antecedent volumes of this series having been mentioned in this department, we deem it unnecessary to do more than announce the advent of Vols. IX. and X. That William Wood & Company could produce this library of twelve volumes at $1.33 each is a marvel, and physicians would do well to avail themselves of the generous offer.

---

THE HEALTH AND HOME LIBRARY, Published by the Health and Home Publishing Co., Chicago.

A very handsome pamphlet. "library size," published quarterly, at $1.00 per year. It contains 96 pages, is gotten up in beautiful style, in an embossed pink cover, with a handsome title page, and comes to us done up in *tissue paper*. and securely enveloped in a patent envelope, with tapes to tie; a manner of taking care of it during its travels and exposure, quite suggestive of its contents. It contains a lengthy article on the "Education of the Senses," or "clairvoyance, clairaudience and Psychometry explained and taught, by Alpha." A novelette entitled "Can you Blame Her," a kind of a temperance story very commendable—in the direction of preservation of home and health, a long article on the "Evils of Vaccination," revised by Dr. Hale." In light of the manifest and manifold blessings of vaccination we have not had the patience to read this article, but pass on to "The Visona, a universal and natural language," which we are now at intervals trying to digest; more anon. Then there comes a chapter devoted to "Woman and her Sorrows," and a health department; a truly rare and interesting publication.

ON THE PATHOLOGY AND TREATMENT OF GONORRHŒA AND SPERMATORRHŒA. By J. D. Milton, senior surgeon to St. John's Hospital for Diseases of the Skin, London. Octavo, 484 pages. Illustrated. Price, bound in extra muslin, $4. New York: William Wood & Company.

We have examined this work with much interest, believing it would present something new. Well, we were not disappointed. For, although there is nothing absolutely *new* in the treatment of gonorrhœa, the comparison of results of various methods serves to give the reader an idea of their relative value. Three hundred pages on gonorrhœa would seem to be exhaustive, yet it seems to be an abridgement of a former edition. The writer is modest, and less dogmatic in the presentation of his views than teachers generally are, and has regard for the opinions of others. The chapter on spermatorrhœa is intensely interesting, but the electrical contrivance for awakening the patient when an erection comes on almost excites one's risibilities. It is a good book to have, and the general practitioner will find it a storehouse of useful information on a very common ailment.

---

A MANUAL OF THE PHYSICAL DIAGNOSIS OF THORACIC DISEASES, by E. Darwin Hudson, Jr., A. M., M. D., late Professor of General Medicine and Diseases of the Chest in the New York Polyclinic; Physician to Bellevue Hospital, etc. One volume. Octavo. 162 pages. Nearly 100 illustrations. Muslin. Price, $1.50. New York: William Wood & Company.

This is a posthumous work, and has met with a very favorable reception at the hands of the profession, the medical press, as a rule, commending it in complimentary terms. It treats of a branch of medical science which is, as a rule, not thoroughly understood by the general practitioner, and there are many points elucidated which the ablest could study with profit. It is gotten up very neatly, and on good paper. Price, $1.50.

---

VICK'S FLORAL GUIDE FOR 1888 has reached our desk. It is a gem to those who love flowers, and an ornament to any drawing-room. The frontispiece is an exquisite colored plate of the new and now famous "Phenomenal" Fuschia, the flowers of which attain a size of three inches in diameter. Mr. Vick will send this beautiful and useful book to any address, together with a *coupon*, entitling the purchaser to ten cents worth of seeds, flower or vegetable, on receipt of ten cents. *Vick's Monthly Magazine* is indispensable to all floriculturists. The "Guide" is issued only in January of each year.

---

A COMPLETE HANDBOOK OF TREATMENT. By Wm. Aitken, M. D., (Edin.) F. R. S., Edited with notes and additions, by A. D. Rockwell, A. M. M. D.; cloth, pp. 444, New York. E. B. Treat, 771 Broadway, 1887.

This is a handsomely bound and very neatly gotten up book, and consists of a synopsis or resume of the treatment of disease in general, as given in the larger and well known edition of Aitkin. The chief feature in the way of an advantage which the work possesses is the analytical index, by means of which one may find just what is wanted without searching through the chapters. It is a very good *condensation* of the larger *Aitken*, and we suppose will fill a "long felt want," and an Aitken void. Some say it is a rare Treat.

